# From emotional intelligence to suicidality: a mediation analysis in patients with borderline personality disorder

**DOI:** 10.1186/s12888-022-03891-6

**Published:** 2022-03-31

**Authors:** Mohsen Khosravi, Fahimeh Hassani

**Affiliations:** 1 Department of Psychiatry and Clinical Psychology, Baharan Psychiatric Hospital, Zahedan University of Medical Sciences, Zahedan, 9813913777 Iran; 2 General Practitioner, Islamic Azad University, Zahedan Branch, Zahedan, Iran

**Keywords:** Addiction, borderline personality disorder, depression, emotional intelligence, self-esteem, suicidality

## Abstract

**Background:**

Borderline personality disorder (BPD) is a serious mental illness with a high suicidality rate between 40 and 85%. However, little is known concerning psychosocial risk and protective factors associated with suicidal behaviors in this clinical group. The main focus of the present study was on examining the relationship of emotional intelligence (EI) with suicidal behaviors and its mediators (e.g., depression, self-esteem, addiction potential, and disorder severity) among patients with BPD.

**Methods:**

In this cross-sectional study, a total of 220 participants (including 110 patients with BPD and 110 healthy controls) in Zahedan, Iran, were examined using clinical interviewing and self-report measures of EI, suicidal behaviors, depression, self-esteem, addiction potential, and BPD symptom severity. The data were analyzed using SPSS v25.0 software at the significance level of *p* < 0.05.

**Results:**

Our preliminary analysis showed higher levels of EI, depression, and self-esteem in the BPD group in comparison to healthy controls (p < 0.001). Furthermore, our findings showed that higher levels of addiction potential, BPD symptom severity, and depression and lower levels of self-esteem and EI were likely to be related to suicidal behaviors of the BPD group. Our results also supported the overall hypothesis that addiction potential, depression, BPD symptom severity, and self-esteem had a mediating role in the impact of EI on suicidal behaviors in the BPD group.

**Conclusions:**

According to these findings, we have come to believe that training EI possibly plays a directly and/or indirectly potential preventive and therapeutic role in suicidal behaviors among patients with BPD. However, further longitudinal studies must be carried out to clarify the cause and effect relationship between EI, depression, self-esteem, addiction potential, BPD symptom severity, and suicidal behaviors.

## Background

Borderline personality disorder (BPD) is a serious public health problem specified by instability along many domains of life, namely interpersonal relations, emotions, and behavior [1]. Emotion dysregulation, as a key feature and core contributor to BPD, comprises deficits in emotion modulation (i.e., being unable to manage one’s emotions) and emotional vulnerability (i.e., affective instability and high sensitivity to emotional stimuli) so that it does not allow individuals to follow essential goals or have an effective behavior in diverse contexts [2, 3].

Early evidence indicated that outpatients with BPD, compared to non-psychiatric controls, have crucial deficits in the important domains of emotional intelligence including self-awareness, control of emotions, motivating oneself, and empathy [4–8]. According to their ability-based model, Mayer and Salovey [9] defined EI as “the capacity of an individual to process emotional information to facilitate social functioning and improve cognitive activities.” As claimed in this model, EI comprises four abilities: perceiving, using, understanding, and managing emotions [10]. Since it has been theorized that emotion dysregulation drives all the symptoms of BPD, poor emotion management is likely to underlie all BPD criteria [3]. Hence, the fifth criterion for BPD (i.e., self-harming/suicidal behaviors) seems to be an abnormal response to emotions and attempts to regulate them [5]. Accordingly, a key part of research and EI theory among patients with BPD in recent years has been devoted to analyzing individual differences in emotion regulation [11]. In this regard, the available research on the development of personality pathology has significantly supported emotion dysregulation as a risk factor for suicidal behaviors among patients with BPD and a fundamental feature of them [8, 12, 13]. As per these recent findings that perceive BPD symptoms to be partly triggered by poor emotion management [3], our prediction was that global BPD, including suicidal behaviors, would be in a negative association with emotional management abilities. However, trait EI might be more intensely implicated since personality disorders are perceived as extreme variants of normal personality traits, including facets of the EI construct [14]. This hypothesis can also be explained by Linehan’s theory. The odds are that trait EI acts analogously to Linehan’s concept of emotional or temperamental vulnerability [3]; it is considered a risk factor for predisposing individuals towards the development of poor emotional functioning and maladaptive BPD traits. Further, there is a significant overlap between certain trait EI facets and BPD traits (e.g., trait emotion management and BPD affective instability). This overlap has consistency with the DSM-5, which partly characterizes BPD as poor affect regulation, implying that BPD symptoms (such as suicidal behaviors) might be conceptualized in terms of poor trait EI [5]. Also, parallels can be found between the emotion management component of ability EI and Linehan’s concept of emotion modulation; both involve abilities to prevent self-destructive behavior associated with strong positive or negative emotions [3, 9].

According to these core concepts and theoretical frameworks, people with higher levels of EI are assumed to regulate their emotions better than people with lower levels of EI [11]. This argument agreed closely with the hypothesis about an association between suicidal behaviors and EI and the latter’s role as a protective factor. Besides, preceding studies identified the association between low levels of EI and four major risk factors in suicidality, i.e., depression, poor self-esteem, high addiction potential, and high severity of BPD symptoms, among non-clinical samples [15–21]. As an illustration, recent studies evaluating the association of depression with emotion perception and social cognition have demonstrated that the problematic social interaction in major depressive disorder may be caused by a moderate and stable deficit in decoding emotional stimuli and mental states [22]. The ability EI model provides a wide framework to analyze the relationship of the emotional ability deficits in major depressive disorder with social and mental health indicators. Emotional skills are encompassed in this framework for perceiving emotions, as well as the ability to understand, use, and regulate emotions [15]. Based on these collective literature reviews, we assumed that the protective capacity of EI in suicidal patients with BPD might be related to its negative correlation with these four major risk factors for suicide. Nevertheless, research on EI in clinical groups, particularly patients with BPD, is still very limited and has hardly grown over time. The limited empirical literature addressing the association between EI and BPD symptoms exhibits an inconsistent picture. A recent study by Beblo et al. [23] failed to reveal any deficits in EI among patients with BPD. Instead, Hertel et al. [24], Avarzamani et al. [25], and Peter et al. [26] found that EI was significantly impaired in the BPD group compared with both non-patients and patients with other personality disorders.

However, to date, only few studies have examined the association between EI and BPD in a clinical population, in which the mean level of EI among patients with BPD has been compared with that in healthy controls [23–26]. Besides, no study has attempted to investigate the causal pathway from trait EI to suicidal behaviors among patients with BPD. In this regard, the present study attempts to replicate previously reported results and provide a better insight into the psychological risk factors for suicidal behaviors in patients with BPD. Accordingly, the primary focus of the current research is on replicating the studies by Hertel et al. [24], Avarzamani et al. [25], and Peter et al. [26] and comparing the levels of EI, depression, and self-esteem between two study groups, including patients with BPD and healthy controls (Hypothesis 1). We also examine the hypothesis about the relationship between EI and suicidal behaviors among patients with BPD; higher EI would be associated with lower levels of suicidal ideation and attempts (Hypothesis 2). Given that previous studies proposed the association between EI, poor mental health outcomes (e.g., depression, low self-esteem, high addiction potential, and BPD diagnosis), and suicidal feelings [15–21], another hypothesis is that depression, self-esteem, addiction potential, and BPD symptom severity would be particularly important mediators of the relationship between EI and suicidal behaviors among patients with BPD (Hypothesis 3).

## Methods

### Study design, setting, and participants

This cross-sectional study was conducted between April and September 2019 in Zahedan, Iran. In this study, 110 patients with BPD and 110 healthy people were selected. The G*Power software version 3.1.9.4 was used to compute the sample size of the study. The sample size for 80% power (considering α error probability of 0.05, a medium effect size of 0.16, and 10 predictor variables) was estimated at 110 people [27]. In addition, a sampling error of 0.1% was obtained, which suggests that sufficient numbers of samples were taken [28]. The patients with BPD were selected by systematic random sampling from among the patients who referred to Baharan psychiatric hospital in Zahedan, Iran, with a sampling interval of 3. Also, the healthy group was recruited from residents of the same geographical area through one-to-one matching. Sampling was continued until reaching 220 completed questionnaires (including 110 patients with BPD and 110 healthy controls). The inclusion criteria were as follows: (i) getting a score above 10 in Borderline Personality Inventory (BPI) [29, 30] and approved diagnosis of the disorder based on Structured Clinical Interview for DSM-5 Personality Disorders (SCID-5-PD) [31] by an expert psychiatrist; (ii) age range of 20–40 years; (iii) ability to read and write alongside reading comprehension; (iv) for the healthy group, getting a score of < 21 in the 28-item General Health Questionnaire (GHQ-28) [32, 33] and approved mental health based on Structured Clinical Interviews for DSM-5 (Diagnostic and Statistical Manual of Mental Disorders, fifth Edition): Clinical Version (SCID-5-CV) [34] by an expert psychiatrist. Exclusion criteria comprised (i) intellectual disability; (ii) a history of neurological disorder; (iii) hearing loss; (iv) failing to fill the questionnaires properly. The socio-demographic information of the participants is presented in Table 1 [*N* = 220, M_age_ = 30.08, SD_age_ = 5.45, Males = 132 (60%), Females = 88 (40%)].

**Table 1:** Socio-demographic characteristics in two study groups (*N* = 220)

Variables	Categories	Healthy Group (*n* = 110)	BPD Group (*n* = 110)	
		M (SD)/ n (%)	M (SD)/ n (%)	Test/p-value
Age		29.88 (5.54)	30.29 (5.38)	t = −0.55 *p* = 0.579
Gender	Male	64 (58.2)	68 (61.8)	χ^2^ (1) = 0.30 *p* = 0.582
	Female	46 (41.8)	42 (38.2)	
Marital status	Single	46 (41.8)	43 (39.1)	χ^2^ (2) = 0.17 *p* = 0.914
	Married	52 (47.3)	54 (49.1)	
	Divorced	12 (10.9)	13 (11.8)	
Education Level	Non-degree	54 (49.1)	59 (53.6)	U = 5693.00 *p* = 0.398
	High school diploma	44 (40)	43 (39.1)	
	Academic degree	12 (10.9)	8 (7.3)	
Income (monthly)	< 15,000,000 Rials	78 (70.9)	85 (77.3)	U = 5665.00 *p* = 0.283
	≥ 15,000,000 Rials	32 (29.1)	25 (22.7)	

Statistical analyzing applied independent t-test, Mann-Whitney U test, and chi-squared test

*BPD* Borderline Personality Disorder

### Procedures

The study was approved by the ethics committee of the Medical Faculty of the Zahedan University of Medical Sciences (IR.ZAUMS.REC.1397.503). After providing essential information about the study’s objectives for the participants and obtaining informed consent from them, an expert psychiatrist evaluated all of the participants using BPI [29, 30], SCID-5-PD [31], GHQ-28 [32, 33], and SCID-5-CV [34] to identify the two study groups (including BPD group and healthy group). Next, socio-demographic information form, Beck Depression Inventory (BDI-II) [35], Self-Esteem Inventory (SEI) [36], and Trait Meta-Mood Scale (TMMS) [37, 38] were given to two study groups. The BPD group was also assessed using Addiction Potential Scale (APS) [39, 40], Borderline Personality Disorder Severity Index (BPDSI) [41], and Beck Scale for Suicidal Ideation (BSSI) [42]. To follow the Helsinki declaration [43], the individuals were told that their participation was voluntary and could leave the study for any reason. All participants were also assured of ethical principles of confidentiality.

### Measures

#### APS

The tendency towards substance use was assessed by the Persian version of the APS, a self-report 36-item questionnaire scored based on a four-point (0–3) Likert scale with the minimum and maximum scores of 0 and 108, respectively [39]. The reliability and validity of APS have been reported to be suitable by Zargar et al. [40]. In our study, the Cronbach’s alpha coefficient for the APS was 0.92.

#### BDI-II

Depressive symptoms were evaluated using the Persian version of the BDI-II. It is a self-report 21-item questionnaire scored based on a four-point (0–3) Likert scale, with the minimum and maximum scores of 0 and 63, respectively [35]. In our study, the Cronbach’s alpha coefficient for the BDI-II was 0.89.

#### BSSI

Suicidal behaviors were evaluated using the BSSI, a self-report, 19-item questionnaire. Each item of this questionnaire is scored based on an ordinal scale from 0 to 2, giving a total score of 0 to 38. The Persian version of BSSI has shown satisfactory psychometric properties [42]. In our study, the Cronbach’s alpha coefficient for the BSSI was 0.90.

#### TMMS

The TMMS was used to evaluate the EI of patients with BPD. This scale consists of 30 items and three subscales, including attention to feeling, clarity in discrimination of feeling, and mood repair [37]. Each question on this scale is scored on a Likert scale from 1 to 5. Therefore, the minimum and maximum possible scores would be 30 and 150, respectively. In Iran, the reliability and validity of TMMS have been reported suitable by Ramezani and Abdullahi [38]. In our study, the Cronbach’s alpha coefficient for the TMMS was 0.82.

#### BPDSI

Borderline personality symptomatology was assessed using BPDSI. This questionnaire included 70 questions to examine 9 criteria for BPD, including abandonment, relationships, identity disturbance, impulsivity, parasuicide, affective instability, emptiness, anger-control, and dissociation. Appropriate validity and reliability have been reported for the Persian version of BPDSI [41]. In our study, the Cronbach’s alpha coefficient for the BPDSI was 0.85.

#### RSE

RSE was used to assess individual self-esteem. This self-report questionnaire includes 10 general statements scored on a 4-point Likert scale from “strongly agree” (3) to “strongly disagree” (0) (and inversely scored for some items) with a total score of 0–30. In Iran, the reliability and validity of RSE have been reported suitable by Salsali and Silverstone [36]. In our study, the Cronbach’s alpha coefficient of the RSE total score was obtained of 0.88.

#### BPI

The BPI was used for the initial screening of patients with BPD. In this 53-item yes/no questionnaire, if the person’s score for the 20 items of the cutoff score is above 10, the person is most likely to be affected by BPD [29, 30]. Reliability and validity of the Persian version of BPI were reported suitable by Mohammadzadeh [30]. In our study, the Cronbach’s alpha coefficient for the BPI total scale was 0.88.

#### SCID-5-PD

The SCID-5-PD was used to confirm the diagnosis of BPD. It is a structured clinical interview used by researchers and clinicians, which evaluates DSM-5 personality disorders (under three clusters of A, B, and C) and other specific personality disorders. Several studies have reported acceptable reliability and validity of SCID-5-PD [31].

#### SCID-5-CV

The SCID-5-CV was used to rule out psychiatric disorders among healthy controls and to identify the history of suicide attempts and SUDs in patients with BPD. It is a structured interview for major DSM-5 diagnoses, which is performed by a trained clinician or health expert familiar with the diagnostic criteria and classification of disorders in DSM-5. Several studies have reported acceptable reliability and validity of SCID-5-RV [34].

#### GHQ-28

Screening of healthy controls was performed using the GHQ-28, first developed by Goldberg in 1978. This 28-item questionnaire is scored on a 4-point Likert scale (0–3), i.e., a total score of 0–84 [32]. In Iran, Taghavi [33] reported the Cronbach’s alpha coefficient of the total score of GHQ-28 to be 0.93. In our study, Cronbach’s alpha coefficient was found to be 0.90.

### Statistical analysis

There was no missing data on any of the scales administered. Firstly, SPSS v25.0 software was used to assess the normality of data, and the descriptive statistical analysis was performed to evaluate the mean scores of each variable. According to the acceptable range for normality in the formal normality tests (including Shapiro-Wilk test and Kolmogorov-Smirnov test), the independent t-test, Mann-Whitney U test, and chi-squared test were used for a socio-demographic comparison among the study groups. Independent t-test was also carried out to compare the mean scores of TMMS, SEI, and BDI-II among two study groups (Hypothesis 1). Next, Pearson correlation coefficient and multiple regression analysis (enter method) were conducted to investigate the relationship between dependent variables (i.e., suicidal behaviors) and independent variables (i.e., EI, depression, self-esteem, addiction potential, BPD symptom severity) in the BPD group (*n* = 110; Hypothesis 2). A mediation analysis with bootstrap sampling was also implemented to evaluate the mediating roles of depression, self-esteem, addiction potential, and BPD symptom severity in the relationship between EI and suicidal behaviors (by controlling the effect of socio-demographic factors, including age, gender, marital status, education level, and income; Hypothesis 3). As per the recommendation of Preacher and Hayes [44], the bootstrap method (with 5000 bootstrap samples and 95% bias-corrected confidence intervals) was applied to estimate direct, indirect, and total effects. All paths were estimated via ordinary least squares regression. These analyses were performed using the Hayes’ PROCESS macro method v3.4.1, a computational procedure for SPSS [45]. As posited by Preacher and Hayes [44], the mediating role is present if the indirect effect is significant and the confidence interval (CI) excludes a zero value. Moreover, only independent variables with a statistically significant correlation with suicidal behaviors among patients with BPD were included in the mediation analysis.

## Results

### Preliminary analysis

**Fig. 1:**
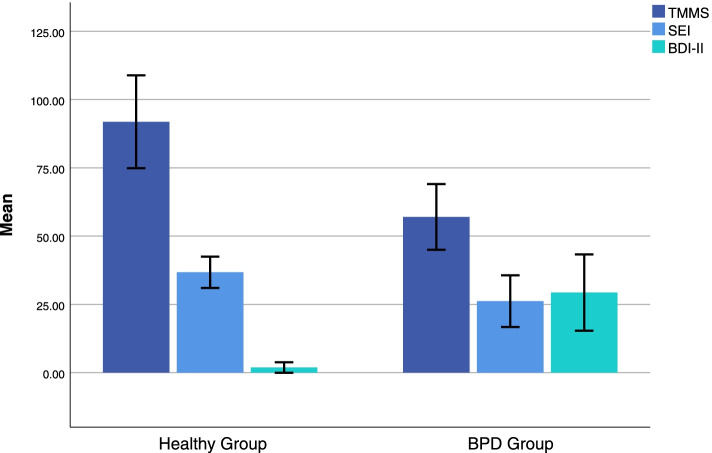
Clustered bar mean of Trait Meta-Mood Scale (TMMS), Self-Esteem Inventory (SEI), and Beck Depression Inventory (BDI-II) by groups (Error bars: 95% CI, ± 1 SD; *N* = 220). *Note*. Statistical analyzing applied independent t-test: TMMS: t = 17.53; df = 196.36; p < 0.001. SEI: t = 10.03; df = 179.47; p < 0.001. BDI-II: t = −20.40; df = 113.14; p < 0.001

In this study, comparisons of mean TMMS, SEI, and BDI-II scores revealed significant differences among the two study groups, including healthy group and BPD group (t = 17.53; df = 196.36; p < 0.001; t = 10.03; df = 179.47; p < 0.001; t = −20.40; df = 113.14; p < 0.001, respectively; see Fig. 1).

Moreover, The results obtained from the correlation matrix in BPD group revealed that the suicidal behaviors had a positive and significant correlation with the depression (*r* = 0.76, *p* < 0.001), addiction potential (*r* = 0.78, *p* < 0.001), BPD symptom severity (*r* = 0.64, *p* < 0.001), and negative and significant correlation with TMMS (*r* = −0.66, *p* < 0.001) and SEI (*r* = −0.61, *p* = 0.003; see Table 2).

**Table 2:** Correlation matrix of study variables among patients with borderline personality disorder (*n* = 110)

Variables	TMMS	SEI	BDI-II	APS	BPDSI	BSSI
TMMS	–					
SEI	0.66^***^	–				
BDI-II	−0.49^***^	−0.40^***^	–			
APS	−0.52^***^	−0.46^***^	0.85^***^	–		
BPDSI	−0.56^***^	−0.55^***^	0.50^***^	0.50^***^	–	
BSSI	−0.66^***^	−0.61^***^	0.76^***^	0.78^***^	0.64^***^	–

Statistical analyzing applied Pearson correlation coefficient

*APS* Addiction Potential Scale, *BDI-II* Beck Depression Inventory, *BPDSI* Borderline Personality Disorder Severity Index, *BSSI* Beck Scale for Suicidal Ideation, *SEI* Self-Esteem Inventory, *TMMS* Trait Meta-Mood Scale

^*^p < 0.05; ^**^p < 0.01; ^***^p < 0.001

### Risk factors related to suicidal behaviors among patients with BPD

Table 3 summarizes the results of multiple regression analysis obtained by enter method. According to the table, EI (β = −0.18, *p* = 0.009), self-esteem (β = −0.18, *p* = 0.010), depression (β = 0.29, *p* = 0.002), addiction potential (β = 0.25, *p* = 0.009), and BPD symptom severity (β = 0.17, *p* = 0.007) could account for 78% of the suicidal behaviors variance among patients with BPD (F (10, 99) = 35.04, p < 0.001). However, no significant relationship was observed between suicidal behaviors and socio-demographic factors such as age, gender, marital status, education level, and income. These findings demonstrated that lower levels of EI and self-esteem and higher levels of depression, addiction potential, and BPD symptom severity might be associated with suicidal behaviors among patients with BPD. Among these variables, depression levels showed the most significant association with suicidal behaviors in patients with BPD.

**Table 3:** Associated factors with suicidal behaviors among patients with borderline personality disorder (*n* = 110)

					95% Confidence Interval	
Explanatory variables	B	SE	β	t	Lower Bound	Upper Bound
Age	−0.88	0.47	−0.09	−1.85	−1.83	0.06
Gender	−0.62	0.91	−0.03	−0.68	−2.43	1.18
Marital status	−0.42	0.66	−0.03	−0.63	−1.73	0.89
Education Level	0.74	0.72	0.05	1.01	−0.70	2.18
Income	−0.48	1.10	−0.02	−0.44	−2.67	1.70
TMMS	−0.14^**^	0.05	−0.18	−2.68	−0.24	−0.03
SEI	−0.17^*^	0.06	−0.18	−2.64	−0.30	−0.04
BDI-II	0.19^**^	0.06	0.29	3.11	0.06	0.31
APS	0.17^**^	0.06	0.25	2.51	0.04	0.31
BPDSI	0.18^**^	0.06	0.17	2.76	0.05	0.31
R	0.883					
R^2^	0.780					
F (df1, df2)	35.04 (10, 99)^***^					

*APS* Addiction Potential Scale, *BDI-II* Beck Depression Inventory, *BPDSI* Borderline Personality Disorder Severity Index, *BSSI* Beck Scale for Suicidal Ideation, *SE* Standard Error, *SEI* Self-Esteem Inventory, *TMMS* Trait Meta-Mood Scale

^*^p < 0.05; ^**^p < 0.01; ^***^p < 0.001

### Mediation analysis

**Fig. 2:**
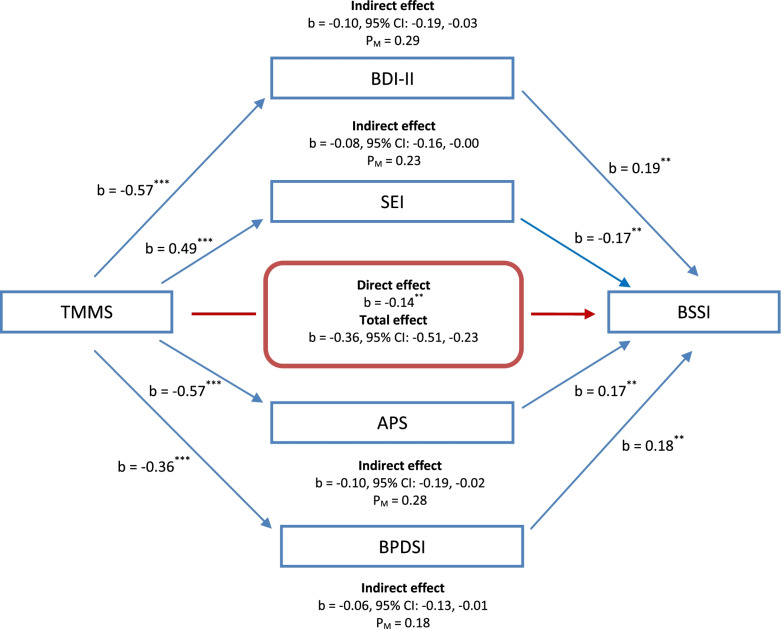
Illustration of the results of the mediation analysis described in the text, which tested BDI-II, SEI, APS, and BPDSI total scores (the measures of depression, self-esteem, addiction potential, and BPD symptom severity, respectively) as the potential mediators of the relationship between TMMS and BSSI total scores (the measures of emotional intelligence and suicidal behaviors, respectively) by controlling for socio-demographic characteristics (including age, gender, marital status, education level, and income) among patients with BPD (*n* = 110). *Note.* APS: Addiction Potential Scale; BDI-II: Beck Depression Inventory; BPD: Borderline Personality Disorder; BPDSI: Borderline Personality Disorder Severity Index; BSSI: Beck Scale for Suicidal Ideation; CI: Confidence Interval; SEI: Self-Esteem Inventory; TMMS: Trait Meta-Mood Scale. P_M_: Effect size (ratio of indirect to total effect). ^*^p < 0.05; ^**^p < 0.01; ^***^p < 0.001

The mediation analysis was conducted as what follows. Initially, the direct effect of predictor variable (i.e., EI, depression, self-esteem, addiction potential, and BPD symptom severity) was examined on the dependent variable (i.e., suicidal behaviors), where all standardized coefficients were significant (see Fig. 2). The mediating effects of depression, self-esteem, addiction potential, and BPD symptom severity on the relationship between EI and suicidal behaviors were also studied to determine statistical significance using Hayes’ PROCESS tool for SPSS (model = 4, bootstrap samples = 5000). Figure 2 represents the indirect effects and relevant 95% CI, as well as EI that had a remarkably direct effect on suicidal behaviors (b = −0.14, *p* = 0.008). On the other hand, depression (b = 0.19, *p* = 0.002), self-esteem (b = −0.17, *p* = 0.009), addiction potential (b = 0.17, *p* = 0.009), and BPD symptom severity (b = 0.18, *p* = 0.006) were also prominent. The indirect effects of EI on suicidal behaviors through depression (b = −0.10, 95% CI: −0.19, −0.03), self-esteem (b = −0.08, 95% CI: −0.16, −0.00), addiction potential (b = −0.10, 95% CI: −0.19, −0.02), and BPD symptom severity (b = −0.06, 95% CI: −0.13, −0.01) accounted for 29, 23, 28, and 18% of the total effect, respectively. Hence, the overall hypothesis 3 that depression, self-esteem, addiction potential, and BPD symptom severity mediated the effect of EI on suicidal behaviors among patients with BPD was supported.

## Discussion

This cross-sectional study pursued three main objectives: (i) comparing the levels of EI, depression, and self-esteem in two study groups including patients with BPD and healthy controls (Hypothesis 1); (ii) exploring the associated factors of suicidal behaviors (Hypothesis 2); (iii) investigating the mediating roles of depression, self-esteem, addiction potential, and disorder severity in the relationship between EI and suicidal behaviors among patients with BPD (Hypothesis 3).

When addressing Hypothesis 1, the present study revealed that the mean TMMS scores among patients with BPD were significantly lower than that among healthy controls. This result was in line with the findings of Hertel et al. [24], Avarzamani et al. [25], and Peter et al. [26], and inconsistent with those of Beblo et al. [23]. However, the sample size of 20 patients in the Beblo et al. [23]‘s study was very small and included only patients with BPD suffering from a moderate degree of severity. However, like the samples of Hertel et al. [24], Avarzamani et al. [25], and Peter et al. [26], our patients with BPD also showed higher severity levels of the disorder. Besides, in agreement with the results of previous studies, higher scores on depression and lower levels of self-esteem were not unexpected in patients with BPD compared to healthy controls [46–49]; even the lability and intensity mean scores of depression among patients with BPD were found to be higher than those of patients with other personality disorders through analyses that controlled for age, sex, and other major affective disorders [50].

When addressing Hypothesis 2, the present study demonstrated that suicidal behaviors among patients with BPD were negatively associated with EI and self-esteem but positively with levels of depression, addiction potential, and BPD symptom severity. Among these variables, depression level was the strongest independent factor associated with suicidal behaviors among patients with BPD. These findings are commensurate with existing literature on risk factors of suicidal behaviors in patients with BPD [51–53]. However, no association was found between suicidal behaviors and socio-demographic characteristics (including age, gender, marital status, education level, and income), which was inconsistent with the studies of Hutsebaut and Aleva [51] and Söderholm et al. [52]. This inconsistency among our findings and previous studies might be attributed to different population groups and cultural expectations. Nonetheless, the specificity of the EI protective effect observed for suicidal behaviors in the BPD group was a great strength of this study. This result agrees with the theory of Linehan [3] and previous results obtained by Hertel et al. [24], showing the impairment in the emotion regulation as an essential feature of the BPD group. This finding has also been supported by cognitive behavioral therapy and dialectical behavioral therapy contributing to improving emotion regulation skills and decreasing suicidality in the BPD group in clinical settings [54]. Contrary to the theory of Linehan [3] and findings obtained by Hertel et al. [24], Peter et al. [26] indicated a central impairment in the emotion understanding ability within the assessment of 61 patients with BPD. They also implied that low emotion regulation ability is a feature of high levels of BPD symptoms in distress rather than a permanent feature of BPD. Based on these inconsistent results, further research is needed to be urgently conducted on the causal relationship between different components of BPD symptoms and EI. However, these findings highlighted the necessity of the patient’s capabilities to understand and manage their emotions when preventing and treating suicidal behaviors. Although previous studies on suicide therapies for patients with BPD have not directly addressed the protective effect of EI, cognitive behavioral therapy and dialectical behavioral therapy (e.g., problem-solving skills or emotion regulation) use the same structures as those examined by EI researchers [18].

When addressing Hypothesis 3, our results indicated that depression, self-esteem, addiction potential, and BPD symptom severity had mediating roles in the relationship between EI and suicidal behaviors among patients with BPD. To explain the above findings, recent evidence has shown that individuals with high levels of EI are able to be aware of their own feelings and those of others. They are also open to positive and negative aspects of internal experience, labeling them, and if necessary, communicating them. Such awareness will often give rise to the effective affect regulation within themselves and others and come up with well-being [55]. Contrarily, many adjustment problems may be due to deficits in EI. Individuals who are unable to understand and recognize the emotions of others or make people feel bad might be perceived to be unpleasant and abnormal and finally ostracized from the community. Moreover, overlooked facets of personal emotions are probably accompanied by planning inability for everyday life and incapability to meet basic emotional needs. Such deficits in planning are likely to provoke depression, unrewarded life, and/or even suicidality [56]. According to recent evidence, low levels of EI might be related to greater BPD symptoms, poor management of emotions, increased risk of addiction, and poor impulse control, all recognized as essential risk factors for suicide [19–21, 57, 58].

### Limitations

This study suffers from a few limitations. As the first restriction, we had a relatively small sample; in other words, our findings were on the basis of a limited sample of episodes of suicidal behaviors. Further, our sample mainly included Baluch ethnic group patients willing to participate in a research study. Accordingly, our findings could not be easily generalized, and it is necessary to replicate them in a more diverse and larger sample. Second, a definite reason cannot be specified behind a correlation by cross-sectional studies in most cases, avoiding a profound understanding of the essence of the causal relationship between study variables. Third, self-report measures were used to report emotions and suicidal behaviors. Therefore, it is feasible that participants mistakenly reported or recalled their emotions and suicidal behaviors. These restrictions can be resolved by designing longitudinal studies and interviewing individual participants. Fourth, the present study did not control for other psychological factors (e.g., intelligence quotient) that might have a contribution to the EI effect, while controlling for them may have strengthened our findings.

Given these limitations, our results should be assumed as preliminary evidence for the EI protective effect, which enhanced our understanding of suicidal behaviors and raised critical issues to be addressed in future research. First, these findings need to be replicated among a more diverse and larger sample, which would support their generalizability. Second, studies should test whether EI can protect against the impacts of co-occurring stressors (e.g., poor-functioning family). As an essential step to clarifying the nature of relations identified here, the degree of generalizability to other stressors and clinical risk factors should be tested. Third, it is worth testing how EI works with other potential protective factors (e.g., social support) to decrease suicide risk. Further, research needs to test the relations between EI and potential genetic and neurobiological predispositions to suicidal behaviors [59–61]. Identifying additional protective factors and developing better theoretical and empirical models would inhibit these prevalent and harmful behavior problems among patients with BPD. Fourth, so far, very few studies have addressed interventions designed to enhance EI in mental disorders. Also, most of these studies had samples of students and, in turn, their findings cannot be applied to clinical populations. However, many psychotherapeutic approaches (e.g., dialectical behavior therapy, transference-focused psychotherapy, mentalization-based psychotherapy, and schema-focused therapy) have been offered to help improve the abilities to recognize, understand, and regulate emotions [26, 41]. Future research needs to examine could these therapies act as a potential mechanism of change. Finally, future studies should focus on the differentiation between positive and negative emotions and assessment of EI within a certain period.

## Conclusions

A naturally arisen, unanswered question is whether suicidal behaviors can be treated or prevented in the BPD group by enhancing EI? Since our findings illustrated that high levels of EI have been directly and indirectly (likely by increasing self-esteem and reducing BPD symptom severity, addiction potential, and depression) related to suicidal behaviors, we conclude that the answer to the above question would be “yes.” Accordingly, training EI probably plays a directly and/or indirectly potential role in treating or preventing suicidal behaviors in the BPD group. However, further longitudinal studies should be carried out to shed light on the probable cause and effect relationship between EI, self-esteem, addiction potential, depression, and BPD symptom severity and suicidal behaviors.

## Data Availability

The datasets generated and analyzed during the current study are not publicly available because no consent was obtained from the participants in this regard. However, the data are available from the corresponding author on a reasonable request.

## Abbreviations

APS: Addiction Potential Scale
BDI-II: Beck Depression Inventory
BPD: Borderline personality disorder
BPDSI: Borderline Personality Disorder Severity Index
BPI: Borderline Personality Inventory
BSSI: Beck Scale for Suicidal Ideation
CI: Confidence Interval
EI: Emotional intelligence
GHQ-28: The 28-item General Health Questionnaire
SCID-5-CV: Structured Clinical Interviews for DSM-5: Clinician Version
SCID-5-PD: Structured Clinical Interview for DSM-5 Personality Disorders
REC: Research Ethics Committee
SEI: Self-Esteem Inventory
TMMS: Trait Meta-Mood Scale
